# Small Bowel Diverticulosis and COVID-19: Awareness Is the Key: A Case Series and Review of the Literature

**DOI:** 10.3390/medicina60020229

**Published:** 2024-01-29

**Authors:** Petros Bangeas, Nikolaos Konstantinidis, Tania Chrisopoulou, Despoina Karatzia, Alexandros Giakoustidis, Vasileios N. Papadopoulos

**Affiliations:** 1 1st University Surgery Department, Papageorgiou Hospital, Aristotle University of Thessaloniki, 54124 Thessaloniki, Greece; nikos4kanti@yahoo.gr (N.K.); despoinakaratzia@hotmail.com (D.K.); alexgiakoustidis@gmail.com (A.G.); papadvas@auth.gr (V.N.P.); 2 Department of Radiology, Genesis General Clinic, 54301 Thessaloniki, Greece; chrysopouloutania@gmail.com

**Keywords:** small bowel non-Meckelian, diverticulitis, COVID-19

## Abstract

Small bowel non-Meckelian diverticulosis is a rare condition with only a few published cases despite being described over 200 years ago. In the midst of the COVID-19 pandemic, studies suggested that many patients may experience gastrointestinal manifestations. Intestinal symptoms could worsen the inflammation and infection associated with small bowel diverticulitis. Here we present three cases: one with inflammation and rupture in a COVID-19 patient and another as an asymptomatic detection. The third case involved recurrence after the first laparoscopic lavage approach. Furthermore, we provide a mini-review of the literature to emphasize the importance of considering this entity in the differential diagnosis of an acute abdomen. In the majority of cases involving small bowel diverticula, conservative management is the preferred approach. However, when complications arise, surgical intervention, including enteroctomy and primary anastomosis, may be necessary to achieve optimal outcomes.

## 1. Introduction

Small bowel non-Meckelian (SBNMD) diverticulosis is an uncommon disorder with a prevalence ranging from 0.06% to 4% [1,2,3,4]. While diverticula are more commonly found in the colon, they can also occur in the small bowel. It typically occurs in the sixth and seventh decades of life, with a significant majority of 80% being over 40 years old at the time of the diagnosis [4]. Multiple jejunal diverticula, especially in the proximal site, are detected in 80% of cases. The remaining 15% of diverticula are located in the ileum, while the remaining 5% occur in both the jejunum and ileum [5]. Both are usually smaller in size, multiple, and isolated. Meckelian diverticula are congenital abnormalities of the small intestine, but non-Meckelian diverticulitis is not associated with this congenital anomaly [1]. From a pathological point of view, SBNMD are deemed “false”, in contrast to Meckel’s diverticula located on the anti-mesenteric site [1,2]. The wide range of atypical symptoms, such as vague abdominal pain, flatulence, diarrhea, and melaena, in combination with its rare occurrence, can lead to a delayed diagnosis and extreme mortality rates [6,7]. Various imaging modalities can be utilized to aid in the diagnosis of non-Meckelian small bowel diverticulitis. A computed tomography (CT) scan is typically the first-line imaging modality used to evaluate patients with suspected diverticulitis. CT can identify inflamed or perforated diverticula, as well as complications such as abscess formation or bowel obstruction. Magnetic resonance imaging (MRI) may also be used to evaluate non-Meckelian small bowel diverticulitis, particularly in cases where radiation exposure is a concern [4,5,6,7]. In addition to CT and MRI, other imaging modalities such as small bowel follow-through studies or enteroclysis may be utilized to evaluate the extent of diverticulitis and any associated complications. Endoscopic retrograde cholangiopancreatography (ERCP) may also be performed to visualize the small bowel and assess for the presence of diverticula especially in case of duodenum localization [2,3,4,5].

Complications frequently occur with colonic diverticula, which are commonly described. These complications include inflammation, perforation, bleeding, and obstruction. The primary diagnostic tool that we use is abdominal computed tomography (CT) [4].

The pandemic has had a significant impact on the management of diverticulitis cases, with delays in seeking medical attention leading to more severe cases upon presentation to healthcare facilities. This delay can be attributed to various factors, including fear of contracting COVID-19 in a healthcare setting, limited access to medical resources due to the overwhelming burden on hospitals, and the reluctance of individuals to seek medical care for what they perceive as non-emergent conditions. Additionally, the strain on healthcare systems has resulted in the postponement or cancellation of non-urgent elective procedures, including colonoscopies, which are crucial in the diagnosis and management of diverticulitis. As a result, many cases of diverticulitis may have gone undiagnosed or untreated, leading to an increased risk of rupture and its associated complications [4,5,6].

Recent studies have suggested a potential link between colitis, diverticulitis, and COVID-19, indicating that individuals with inflammatory bowel diseases may be at an increased risk of experiencing more severe symptoms and complications if they contract the virus. The reasoning behind this correlation lies in the role of the immune system. Colitis is known to weaken the immune system and predispose individuals to infections. When faced with the SARS-CoV-2 virus, individuals with colitis may struggle to mount an effective immune response, leading to a heightened risk of severe illness and complications associated with COVID-19. Furthermore, patients with colitis often require immunosuppressive medications to manage their condition, which could further compromise their ability to fight off the virus. This highlights the importance of careful management and monitoring of individuals with colitis during the ongoing COVID-19 pandemic. It is crucial for healthcare providers to recognize the potential correlation between inflammatory bowel diseases and COVID-19 and take appropriate measures to protect and support individuals with colitis, such as prioritizing vaccination, closely monitoring symptoms, and providing timely medical intervention when necessary [5,6,7].

The fact is that the exact mechanism has not been determined; however, it appears that the SARS-CoV-2 virus (COVID-19) primarily targets the respiratory tract through its affinity for ACE2 receptors. These receptors are also prevalent in intestinal cells, leading to symptoms affecting both the respiratory and gastrointestinal systems. COVID-19 affects the bloodstream, causing hyperactivity in platelets and cytokine storms. This can result in damage to the gut barrier and changes to the gut microbiota. Additionally, intestinal vessel thrombosis can occur, leading to malabsorption and malnutrition. These negative effects can increase the severity of the disease and have both short- and long-term consequences, including gut inflammation and worsening of inflammatory bowel diseases [8,9]. All these had a significant impact on the management of diverticulitis, leading to an increased risk of rupture and potentially life-threatening outcomes. It is crucial for individuals to seek prompt medical attention for symptoms of diverticulitis and for healthcare systems to prioritize the management of these cases to prevent severe complications.

Management of SBNMD typically includes a combination of conservative and surgical interventions. The specific treatment approach depends on the rupture’s severity, complications, and the patient’s overall condition. Every stable patient with localized inflammation and without any radiologic sign of perforation is treated conservatively with bowel rest and broad-spectrum IV antibiotics. If the patient does not show clinical improvement within 48–72 h or if generalized peritonitis or perforation is detected, an exploratory laparotomy may be necessary, which involves small bowel resection with anastomosis [5,6,7].

Herein, we present three cases of SBNMD. The first case involved a COVID-19-positive patient with rupture, while the second case was discovered incidentally. The third case involved recurrence after the first laparoscopic lavage approach. The cases discussed in this report highlight the need for careful management and monitoring of SBNMD, particularly in high-risk patients such as those with COVID-19. Additionally, these cases underscore the importance of prompt detection and intervention in cases of recurrence. It is hoped that this report will contribute to the existing body of knowledge regarding the diagnosis and treatment of SBNMD and, ultimately, lead to better outcomes for patients afflicted with this condition. In addition, a review with a statistical analysis of all the cases reported in the past literature, discussing the most common clinical features, was performed. Also, we proposed the role of laparoscopic surgery as a therapeutic option in disease management.

## 2. Materials and Methods

A literature review was performed using PubMed, Scopus, and Science Direct. The search terms employed were “small bowel diverticulosis”, “jejunal diverticulitis”, and “Ileus diverticulitis”. Since 2010, 352 articles have been published.

**Scheme 1:**
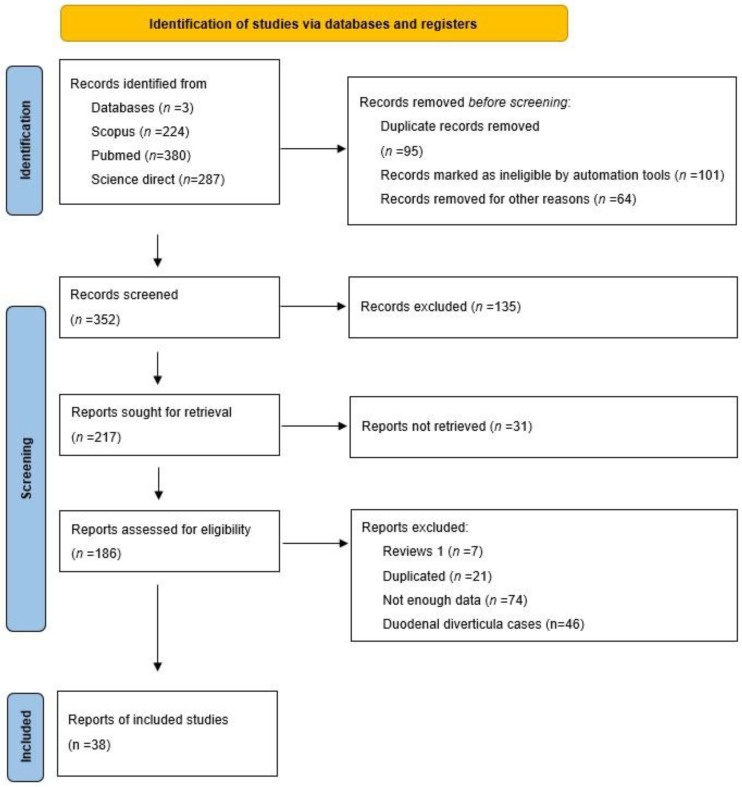
Prisma chart with studies included in our manuscript.

Among these, 217 well-documented papers were identified. There were no restrictions on the ages of the articles included in this review. One hundred eighty-six articles were in English, while 31 were in other languages. All these studies were carefully studied. We had only full texts, case reports, and case series articles in the final assessment. We finally selected 38 articles (41 patients), and a database with the patients’ characteristics was created and is shown in the Prisma chart below (Scheme 1).

The database included sex, age, diverticula location, symptoms, diagnostic methods, treatment management, and complications. The cases that fulfilled all these seven criteria were included in the statistical analysis. Three additional cases were added from the clinical experience of the authors of this article. Thus, a total of 44 patients were included in the statistical analysis. After obtaining ethical approval and participant consent, personal data were removed, and all clinical data were collected. In order to ensure that our study was conducted with the utmost respect for ethical guidelines and research standards, it was crucial to prepare a CARE checklist (Appendix A). Descriptive statistics were used to express the results appropriately. Means, medians, and SDs were used for continuous variables and frequencies for categorical variables. The statistical significance was set at *p* < 0.05. The statistical analysis was performed using SPSS version 25 (SPSS Inc., Chicago, IL, USA).

## 3. Case Report

### 3.1. Case 1

**Figure 1:**
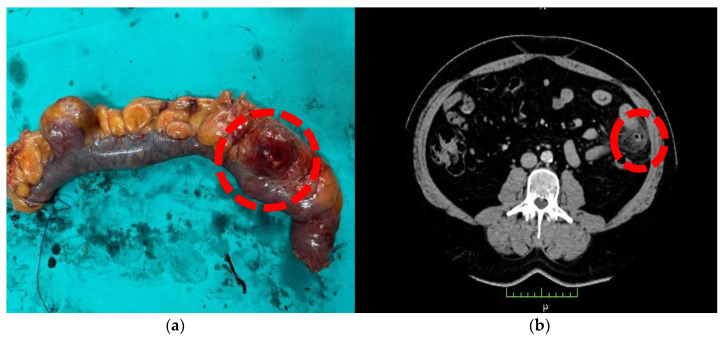
(**a**) Case 1 ruptured jejunum diverticula. (**b**) Abdominal CT, showing ruptured non-Meckelian diverticula. In Circle we can see the intraoperative (**a**) and radiological imaging (**b**) ruptured part of diverticula.

A 45-year-old male presented to our emergency department, complaining about abdominal pain, particularly on the left side, that had been ongoing for 48 h. The clinical examination revealed abdominal distension and tenderness. The laboratory results showed a mildly elevated white blood cell count (9.93 × 10^9^/L) and neutrophilia (88%). During the preoperative examination, it was discovered incidentally that the patient had contracted COVID-19 infection. An emergency CT scan of the abdomen revealed free air, fluid collection in the left abdomen, and two small bowel diverticula in the jejunum with local phlegm (Figure 1a). From the patient’s past medical history, there is no record of diverticula except experiencing upper gastrointestinal bleeding 11 years ago. Additionally, the patient had a history of cardiac arrest and a pacemaker implanted 15 years prior.

Informed written consent was obtained from the patient, and he was taken to the operating theater for an exploratory laparoscopy, where a ruptured part of the jejunum was found (Figure 1b). A partial small bowel laparoscopic enterectomy was carried out with primary side-to-side intracorporeal anastomosis with an endoscopic linear stapler and PDS 2-0 running suture. The patient was discharged from the surgery without drainages and received clear fluids in the afternoon after the surgery. He was mobilized six hours after surgery. Throughout the hospitalization period, he remained stable, and on the third postoperative day, he was discharged. The patient followed a fast-track post-operative protocol (ERAS), which included mobile communication and visits from a specialist nurse.

### 3.2. Case 2

**Figure 2:**
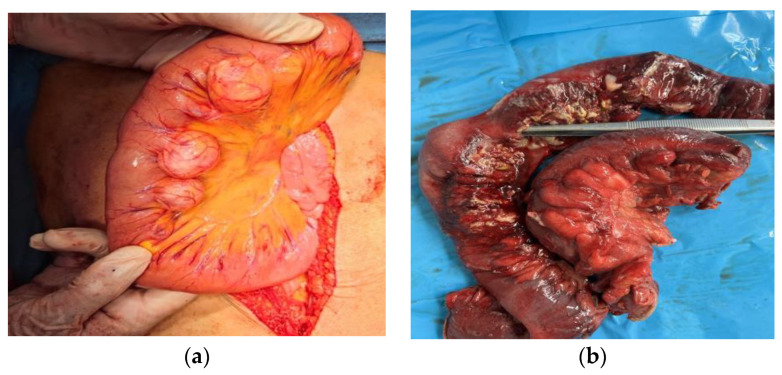
(**a**) Incidentally identified SBNM diverticula. (**b**) A ruptured ileum diverticula with inflammation extension in sigmoid colon.

A 75-year-old male presented to our department due to a rectal tumor of 9 cm from the anal verge. The patient underwent an operating theater for a scheduled low anterior resection. During the operation, multiple jejunal diverticula were identified (Figure 2b). Upon further examination, it was determined that these diverticula were not inflamed, therefore obviating the necessity for their removal. It is worth noting that no diverticula were mentioned in the preoperative CT of the abdomen.

### 3.3. Case 3

Upon presentation, a patient aged 46 years complained of acute abdominal pain, which had persisted for a period of 24 h. The clinical examination revealed abdominal distension and tenderness. The laboratory results indicated an elevated white blood cell count of 16.93 × 10^9^/L and neutrophilia of 90%. During a preoperative examination, it was incidentally discovered that the patient had contracted COVID-19. Subsequently, an emergency computed tomography (CT) scan of the abdomen revealed the presence of free air, fluid collection in the left abdomen, and diverticula in the sigmoid colon with local phlegm (as depicted in Figure 1a). The patient had no significant medical history prior to this incident. In order to investigate the condition further, the patient underwent an investigative laparoscopy in the operating theater. During the procedure, it was observed that there was no presence of fecal content. However, a purulent collection was identified and subsequently drained. Further investigation revealed a small rupture in the ileum diverticula, which was repaired using laparoscopic suturing. It is noteworthy that the rupture was not found in the sigmoid colon. This was followed by laparoscopic lavage and drains placement. The postoperative theater was uneventful. Following surgical intervention, the patient in question received a regimen of antibiotics, specifically meropenem and metronidazole. Despite this treatment, on the third day post-surgery, the patient presented with a fever of 39.1 °C and leukocytosis (27 × 10^9^/L). Consequently, an exploratory laparoscopy procedure was conducted, ultimately exposing a substantial intra-abdominal collection. Following laparoscopic lavage, we determined that the inflamed section of the ileum required an enterectomy and side-to-side anastomosis. Furthermore, the inflammation had significantly corroded the walls of the sigmoid colon, necessitating a partial sigmoidectomy with a hand-sewn end-to-end anastomosis (Figure 2b). The patient was administered clear fluids on the first postoperative day and was mobilized just twelve hours after surgery. Throughout their hospitalization, the patient remained stable and was discharged on the fifth postoperative day after the second surgery.

## 4. Results

After conducting a literature review, we determined the characteristics of jejunal diverticula based on various factors, including sex, age, length, location of the diverticula, significant symptoms, type of procedure, survival rates, and potential complications categorized by Clavien–Dindo classification [2,4,5,6,7,10,11,12,13,14,15,16,17,18,19,20,21,22,23,24,25,26,27,28,29,30,31,32,33,34,35,36,37,38,39,40,41,42,43,44].

Regarding sex, 63.63% were male (28 patients), whereas 36.37% were female (16 patients). The ratio between males and females was 2:1, suggesting a male predominance in the reported population. Our database is shown below (Table 1).

**Figure 3:**
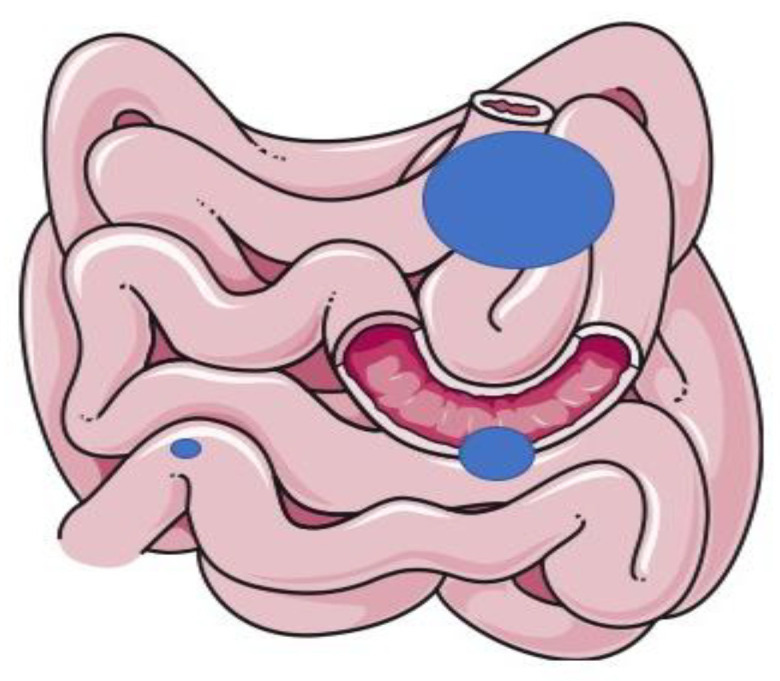
SMNMD localization.

Based on our database (Table 1), it appears that diverticula are typically found in the jejunum, as illustrated in Figure 3 (31 patients, 70.45%), with only 9 patients with diverticula found in the proximal ileus (20.45%) and 4 patients with diverticula in the distal ileus (9.09%).

**Figure 4:**
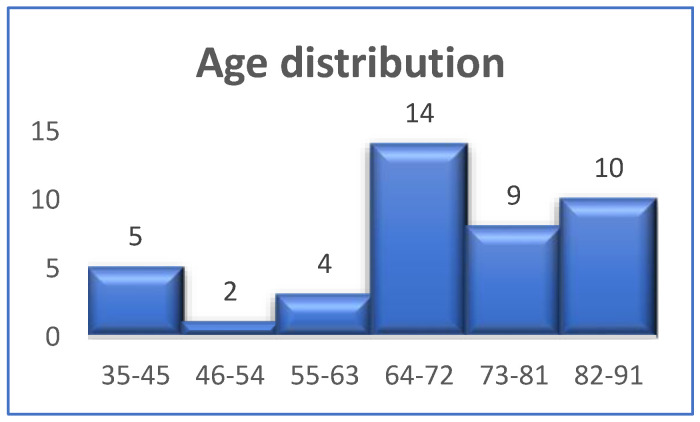
Age distribution.

The age distribution is shown in Figure 4, from which it is concluded that jejunum diverticula most frequently appeared in the age ranges of 64–72, 73–81, and 82–91 years. The median age of the 44 cases (100%) was 71 ± 14.40 years, ranging from 36 to 91 years. The distribution of age among male and female patients was compared, and it was found that the age at which the tumor appeared did not differ statistically between males and females (70.12 ± 13.27 and 70 ± 15.99 years, respectively; *p* = 0.98).

**Figure 5:**
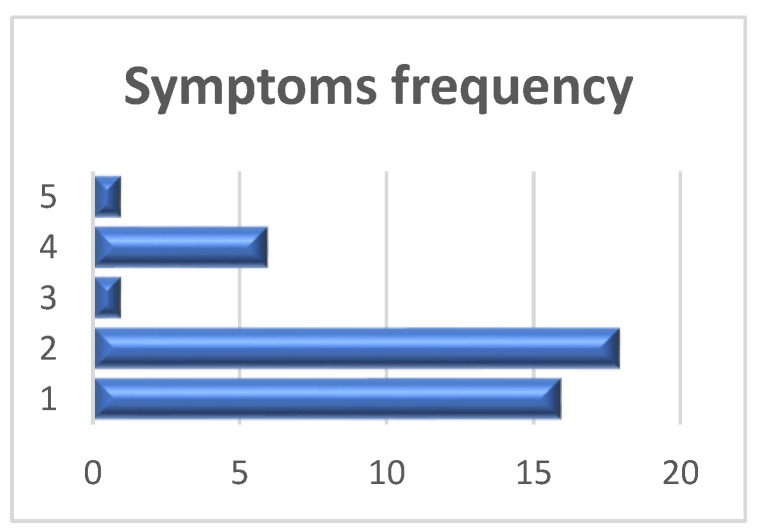
Diverticula symptoms.

The symptoms of SBNMD were also evaluated and are shown in Figure 5. Symptoms were characterized as inflammatory without rupture (1) (16 patients, 36.36%), perforation (2) (19 patients, 43.18%), bleeding (3) (1 patient, 2.27%), (4) obstruction (6 patients, 13.63%), and non-specific/random (5) (2 patients, 4.54%).

Out of 44 patients, only 8 (18.18%) received conservative treatment, while the majority (35 patients, 79.54%) required surgical intervention. Diverticula were discovered accidentally in two patients (4.54%) without signs of inflammation, and no interventions were performed. Of the 44 patients under study, open laparotomy was performed on 33 (75.01%) patients. Conversely, laparoscopic surgery was conducted on only three (6.81%) patients, while the remaining eight patients (18.18%) received conservative care. This indicated that the majority of patients preferred open laparotomy over the laparoscopic approach. The findings underscore the need for further investigation to determine the factors that contribute to patient preferences for open laparotomy and whether laparoscopic surgery is a viable alternative for patients undergoing the procedure.

The median hospitalization was 7 ± 2.37 days, ranging from 3 to 16 days, depending on the treatment option. Hospitalization periods were found to be prolonged for patients who underwent open surgery or were treated using conservative methods.

In order to standardize the complications of our study we use Clavien–Dindo classification, which is a widely used system to categorize surgical complications based on their severity and the treatment required to address them. In accordance with the utilized classification system, it was determined that a total of 29 patients (65.90%) did not necessitate pharmacological, surgical, endoscopic, or radiological interventions (Clavien–Dindo I). Conversely, 12 patients (27.27%) received antibiotic care and parental nutrition (Clavien–Dindo II). Further, two patients (4.54%) required additional surgical procedures (Clavien–Dindo IIIb), while regrettably, a single patient (2.27%) succumbed to their illness during the post-operative period (Clavien–Dindo V).

## 5. Discussion

Jejuno-ileal diverticulitis, a condition involving inflammation of the diverticula in the small intestine, was initially identified by Sommering in 1794 and further researched by Sir Astley Cooper in 1804 [1]. Due to its low prevalence, there have been only a few reported cases since then. The pathophysiology still needs to be fully understood. These are false diverticula of the small intestine, similar to colonic diverticula, where the mucosa and submucosa protrude through the muscular wall. We assume that an elevated intraluminal pressure combined with bowel wall weakness causes these cases. While they are usually asymptomatic, they can lead to life-threatening outcomes when complicated [2,5,8,11].

In the case of uncomplicated SBNMDs, no specific symptoms can lead to the diagnosis. These patients usually complain of abdominal pain, which sometimes radiates in the back, nausea, emesis, fever, and constipation. Due to the greater frequency in the older adult population, where many other health issues or previous surgical history can co-exist, the diagnosis could be challenging [2,5,7,27,28,29,30,31,32,33,34].

Numerous reports have emerged during the COVID-19 period regarding inflammation and bowel ruptures. This is a matter of concern, and we should examine these reports closely to determine the root cause of such incidents and identify measures to mitigate them in the future. Several scientific investigations have highlighted a decline in the number of visits related to diverticular disease during the COVID-19 pandemic. The reduction in visits can be attributed to various factors, including the implementation of social distancing measures, fear of contracting the virus, and limited access to healthcare facilities. However, despite the decrease in overall visits, there has been a significant increase in the prevalence of severe cases. This trend is likely due to a delay in presentation as patients may be reluctant to seek medical attention due to concerns about exposure to the virus. Additionally, there are still instances of COVID-19 patients experiencing a worsening of their diverticular disease, which may be attributed to the effect of the virus on the immune system. Therefore, it is crucial to raise awareness about the risks of delaying medical care and to encourage patients to seek timely treatment to avoid potentially life-threatening complications [35,36,37].

In some studies, it seems that gut enterocytes have been determined to be significant targets of the COVID-19 virus. The virus can enter the cells of the ileum, colon, and esophagus by using Angiotensin-converting enzyme 2 receptors and transmembrane serine protease 2 (TMPRSS2) as a mediator [8,9]. This can lead to gut inflammation and exacerbation of inflammatory bowel diseases, causing negative effects that can worsen the disease severity in both the short and the long term [9].

A comprehensive patient history must be obtained to rule out other conditions such as appendicitis, cholecystitis, colonic diverticulitis, pancreatitis, bowel obstruction, or foreign body perforation [3,4,8,9,10,11,12,13,14,15,16,17,18,19,20,24,34]. An abdominal X-ray can help detect perforation or obstruction but cannot establish a diagnosis on its own. The surgeon must maintain a heightened level of suspicion to ensure the procedure’s accuracy and safety. This involves being vigilant and attentive to any potential risks or complications that may arise during the surgery.

Currently, the gold standard imaging study for abdominal scans still involves using CT scans with both oral and intravenous contrast. Due to the rarity of the disease, there are instances in the literature where the diagnosis was not clearly established before the exploratory intervention but only during exploratory laparoscopy. In our cases, the preoperative CT scan was exact for the site and the number of the ill-defined diverticula [28,29,30,31,32,33,34,35,36,37,38,39,40,41,42,43,44].

As mentioned before, inflammation is the most common complication, followed by perforation. First-line treatment for uncomplicated cases involves IV antibiotics and bowel rest strategies. An annual monitoring protocol, including a clinical examination and CT scan of the abdomen, is necessary in case of accidental detection. Prior history-based clinical suspicion is also important [4,28,35,44,45,46,47,48,49].

**Figure 6:**
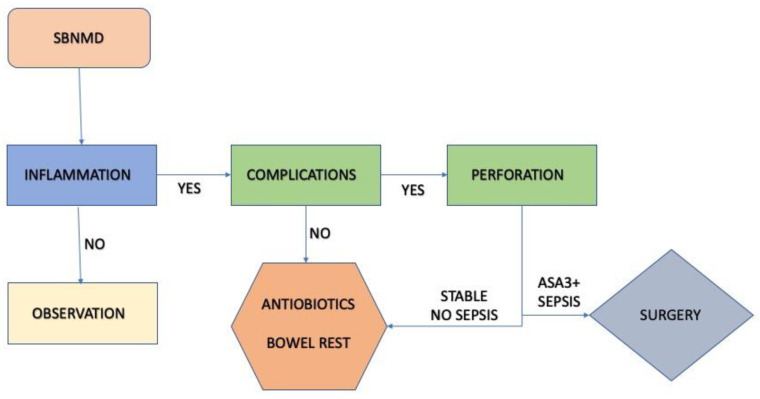
Suggested strategy of SBNMD treatment.

Surgical intervention must be undertaken without delay in cases of sepsis, instability, or severe comorbidities following perforation. These complications could be severe and lead to serious difficulties if not addressed promptly. The type of procedure (open or laparoscopic) does not seem to play an important role and appears to be determined by the surgeon’s preference and center experience. If a patient has a good performance status, surgeons could perform an exploratory laparoscopy and proceed with a conversion procedure (laparotomy) if needed. The length of the bowel resection is also a matter of discussion. There are cases in the literature where the extreme length of bowel disease does not make wide resection feasible [40,47,48,49,50,51]. Most surgeons resect only the complicated diverticular disease and leave the uncomplicated diverticula behind. Based on summarized publications, we create an algorithm in order to manage jejunum diverticulum (Figure 6).

According to our algorithm, in the first case, the patient was hemodynamically stable but had comorbidities (ASA3). The CT scan revealed free abdominal air caused by the perforation of one of two jejunal diverticula. The decision was made to perform surgery on the patient without delay. An exploratory laparoscopy was our first choice, while laparoscopic lavage is seen as an acceptable and safe alternative in selected patients [49]. The patient had no previous abdominal operations and no neglected fasciitis. We recognized the phlegmon and the two complicated diverticula at about 10 cm from each other. An enterectomy was performed with primary anastomosis. The specimen was about 15 cm long, so there was no risk of intestinal failure and small bowel syndrome disease (SBS).

In the second case of accidental detection, we decided on an annual monitoring protocol with clinical examination and CT of the abdomen.

The complexity of our last case was amplified by the dearth of literature available on the subject. While laparoscopic lavage is generally recognized as the gold standard procedure for managing perforated colon diverticulitis, its suitability as a similarly effective and secure option for addressing small bowel diverticula rupture is not yet established. Further research is required to establish the efficacy of laparoscopic lavage in the treatment of these cases. Based on our case and the limited literature on small bowel diverticula, it appears that segmental limited resection with primary anastomosis may be the singular viable option if surgical intervention is necessary.

In both case 2 and case 3, small bowel diverticula were misdiagnosed. These diverticula can be misdiagnosed in radiological examinations due to their small size and the potential for overlap with other structures in the abdomen. One common misdiagnosis of small bowel diverticula is that they are mistaken for small bowel tumors. Due to their similar appearance on imaging studies, such as CT scans and MRIs, small bowel diverticula can be mistakenly identified as tumors, leading to unnecessary biopsies or surgeries. Another potential misdiagnosis is that small bowel diverticula can be overlooked entirely. These tiny pouches may not be easily visible on imaging studies, especially if they are located in obscure areas of the small intestine or if there is poor distention of the bowel during the study. As a result, these diverticula may be missed or dismissed as insignificant findings [48,49,50,51]. The accurate diagnosis of small bowel diverticula on radiological examinations can pose a significant challenge to clinicians. As a result, it is imperative for radiologists to remain cognizant of the potential for a misdiagnosis and to meticulously evaluate any small bowel abnormality to ensure that the correct diagnosis is made and appropriate management is administered to the patient. Thus, it is vital for healthcare providers to exercise due diligence and maintain a high level of vigilance when assessing patients with suspected small bowel diverticula [51].

Furthermore, small bowel diverticula can mimic other conditions such as small bowel obstruction or inflammatory bowel disease. This can lead to a delay in the appropriate diagnosis and treatment of the patient.

## 6. Conclusions

Small bowel diverticula are uncommon but significant medical conditions that require careful monitoring and management. While they may often be asymptomatic, they can lead to serious complications such as bleeding, obstruction, and perforation. Therefore, it is important for surgeons to consider small bowel diverticula as a potential cause of abdominal pain and gastrointestinal symptoms, especially in older patients and in the era of COVID-19.

As we already discussed, the diagnosis of small bowel diverticula can be challenging as they may not always be detected on routine imaging studies. However, advances in imaging technology, such as CT enterography and capsule endoscopy, have improved our ability to identify and characterize these lesions. This, in turn, has led to better understanding of their clinical significance and the potential for intervention.

While surgical intervention has been the traditional treatment approach, further advancement in diagnostic tools may offer less invasive and potentially more effective options. For uncomplicated cases, the management of small bowel diverticula typically involves a combination of dietary modification, symptom control, and surveillance for potential complications.

Strong evidence-based guidelines are needed to guide clinicians in the diagnosis, management, and follow-up of patients with SBNMD. Improving our understanding of this rare condition will lead to better outcomes and quality of life for our patients.

## Figures and Tables

**Table 1:** Diverticula cases publication.

Year	Author	Disease Location	Cases	Age/Gender	Treatment
2017	Marcano	Jejunal	1	71/male	Conservative
2019	Almaki	Jejunal	1	65/Male	Open Surgery
2019	Aispuro	Jejunal	1	86/Male	Open Surgery
2019	Saritas	Jejunal	2	36/Female	Open surgery
		Ileus		75/Female	Open surgery
2020	Ghandour	Jejunal	3	71/Male	Open Surgery
		Jejunal		69/Male	Open Surgery
		Jejunal		55/Male	Conservative
2020	Kunishi	Jejunal	1	41/Male	Conservative
2020	Leigh	Jejunal	1	59/Male	Open Surgery
2020	Ramzee	Jejunal	1	69/Male	Open Surgery
2020	Sammartino	Jejunal	1	91/Male	Open Surgery
2020	Yeung	Jejunal	1	83/Male	Open Surgery
2021	Alyekbeni	Jejunal	1	70/Male	Conservative
2021	Anjum	Jejunal	1	70/Male	Open Surgery
2021	Chung	Jejunal	1	69/Female	Open Surgery
2021	Duggan	Jejunal	1	78/Male	Open Surgery
2021	Giufrida	Jejunal	1	54/Female	Open Surgery
2021	Hardon	Jejunal	1	37/Female	Open Surgery
2021	Khsiba	Jejunal	1	76/Female	Conservative
2021	Kim	Jejunal	1	65/Female	Conservative
2021	Mendo	Ileus	1	73/Male	Open Surgery
2021	Rajaguru	Ileus	1	74/Male	Open Surgery
2021	Vayzband	Jejunal	1	71/Male	Open Surgery
2021	Watanabe	Distal Jejunal	1	72/Male	Open Surgery
2022	Abdelohamil	Jejunal	1	69/Female	Open Surgery
2022	Beti	Jejunal	1	83/Male	Open Surgery
2022	Coelen	Jejunal	1	85/Male	Open Surgery
2022	Eifanagely	Jejunal	1	79/Female	Conservative
2022	Glasser	Ileus	1	57/Male	Open Surgery
2022	Imasato	Jejunal	1	76/Female	Open Surgery
2022	Karna	Jejunal	1	89/Female	Embolization
2022	Lutaya	Jejunal	1	89/Female	Open Surgery
2022	Massoir	Jejunal	1	84/Female	Open Surgery
2022	Matli	Jejunal	1	41/Male	Conservative
2022	Scheesee	Jejunal	1	85/Male	Open Surgery
2022	Pajtak	Jejunal	1	88/Female	Open Surgery
2022	Prough	Jejunal	1	65/Male	Open Surgery
2022	Waiter	Ileus	1	59/Male	Open Surgery

## Data Availability

The datasets used and analyzed during the current study are available from the corresponding author upon reasonable request.

## Abbreviations

SBNMD	Small bowel non-Meckelian diverticulosis
CT	Computed tomography
SPSS	Statistical package for social science software
SBS	Short bowel syndrome
